# Acknowledgement to Reviewers of *Cancers* in 2018

**DOI:** 10.3390/cancers11010065

**Published:** 2019-01-09

**Authors:** Cancers Editorial Office

**Affiliations:** MDPI, St. Alban-Anlage 66, 4052 Basel, Switzerland

Rigorous peer-review is the corner-stone of high-quality academic publishing. The editorial team greatly appreciates the reviewers who contributed their knowledge and expertise to the journal’s editorial process over the past 12 months. In 2018, a total of 545 papers were published in the journal, with a median time to first decision of 18 days and a median time to publication of 38 days. The editors would like to express their sincere gratitude to the following reviewers for their cooperation and dedication in 2018:

Aasen, TrondLi, DeminAbad, CatalinaLi, FuyangAbbas, TarekLi, Hsing-HuiAbburi, ChandrikaLi, HuaAbdel-Hakeem, MohamedLi, LiAbe, HiroyukiLi, LianboAbe, TakanoriLi, LipingAberger, FritzLi, Mei-LingAbeywardana, TharindumalaLi, Shengwen CalvinAccardi, RositaLi, WenliangAdamek, AgnieszkaLi, XiaohongAdamson, David CoryLi, YanAdhikary, AmitavaLi, YvonneAdibi, MehradLian, Christine G.Adighibe, OmanmaLiao, DaiqingAdriaenssens, EricLibra, MassimoAgarwal, AtinLieberman, Paul M.Agelaki, SofiaLiebman, MichaelAggarwal, Bharat B.Lietha, DanielAgodi, AntonellaLim, BoraAgrawal, SwatiLim, Dong JunAhmad, AamirLim, SteveAhmed, Atique U.Lim, Sung-JigAhmed, KhalilLin, Ruo-KaiAhn, Yong ChanLin, Sue-HwaAidinis, VassilisLin, XinAkilov, Oleg E.Lin, YiguangAl Tabaa, YassineLindström, Mikael S.Alaimo, AlessandroLipton, AllanAlcantara, MarionLiss, Andrew S.Aldinucci, DonatellaLittle, Peter J.Alesutan, IoanaLiu, C.M.Alfredo, AddeoLiu, DelongAli, NawabLiu, HaiyanAljabali, AlaaLiu, HongYanAllan, AllisonLiu, JinsongAllegra, EugeniaLiu, SiqiAllen, Steven P.Liu, WeiAlmed, NuzhatLiu, YuAloraifi, FatimaLladser, AlvaroAltomonte, JenniferLloyd, RicardoAmrutkar, ManojLo Muzio, LorenzoAndera, LadislavLo, Hui-WenAnděra, LadislavLo, Kwok WaiAndersen, Jens RikardtLoblaw, AndrewAndrews, LesleyLoessner, DanielaAngelova, Assia L.Löffek, StefanieAngelucci, AdrianoLogue, Susan E.Anguille, SébastienLöhr, MatthiasAntonios, GiakountisLonardo, AmedeoApisarnthanarax, SmithLopez, Jose JavierApostolopoulos, VassoLopez-Crapez, EvelyneArati, SharmaLópez-Guerrero, José AntonioArdolino, MicheleLorberboum-Galski, HayaArena, SabrinaLorenz, KristinaArlt, AlexanderLoskog, AngelicaArnold, MichelleLosurdo, GiuseppeAslam, MuhammadLouie, Alexander V.Aspenström, PontusLowes, Lori E.Attaluri, AnilchandraLozada-Delgado, Eunice L.Aushev, VasilyLu, Jyh-FengAutexier, ChantalLu, YiAveic, SanjaLudueña, RichardAylon, YaelLui, Vivian Wai YanAzer, Samy A.Lukaski, Henry C.Azizi, EbrahimLukong, K. EriqueAzzouz, MimounLumniczky, KatalinAzzouzi, Abdel-RahmeneLuna, Jesus IsaacBaba, HideoLundstrom, KennethBaba, YuhLuo, JiaBach, Duc-HiepLuparello, ClaudioBachanova, VeronikaLuwor, Rodney B.Badawi, MohamedMa, WenBae, Jong-MyonMäättä, Jorma A.Bai, ShuhuaMaayah, ZaidBaiocchi, MartaMace, ThomasBajpai, RichaMackenzie, IanBakht, MartinMacNeill, AmyBaksh, ShairazMadala, Hanumantha RaoBalan, SreeKumarMadenci, Arin L.Balani, JyotiMaggiolini, MarcelloBallarini, FrancescaMahadevan, DarukaBallerini, PatriziaMahajan, AnitaBamford, ConnorMahmud, K. A. FoyezBanciu, ManuelaMai, SabineBanerjee, Hirendra NathMaia, CláudioBanerjee, SusanaMajhen, DragomiraBaran, MarzenaMakena, MonishBaranova, AnchaMale, Victoria H.Barata, JoãoMalhotra, JyotiBarault, LudovicMalhotra, UshaBarbagallo, DavideMalpeli, GiorgioBarbieri, AntonioMancias, Joseph D.Barbolina, MariaMangin, Pierre H.Barrea, LuigiMangla, AnkitBarrero, Maria J.Mantamadiotis, TheoBarrott, JaredMarcato, PaolaBartoszewska, SylwiaMarcel, VirginieBasak, DebasishMarchant, Jonathan S.Bashkin, James K.Marchini, CristinaBasilico, ClaudioMarengo, BarbaraBasolo, FulvioMargit, BalázsBateman, Joesph MatthewMarin, José Juan GarcíaBathula, ChandraMaroni, PaolaBattinelli, Elisabeth M.Marosi, ChristineBaumert, Brigitta G.Márquez Gómez, JavierBaxter, Robert C.Martelli, Alberto MariaBayascas, JoseMartin, Robert C. G.Bazhin, AlexandrMartinelli, GiovanniBeck, WilliamMartinez-Climent, Jose A.Becker, JuergenMartínez-Soler, FinaBeebe, StephenMartinez-Useros, JavierBeg, MuhammadMartin-Fontecha, AlfonsoBeierle, ElizabethMary Ellen, UrickBelletti, BarbaraMary, DidierBellezza, IlariaMast, Mirjam E.Beltran, ChrisMasuelli, LauraBenati, DanielaMasui, KentaBender, DavidMatesic, Lydia E.Benevolenskaya, ElizavetaMatrisian, Lynn M.Beninati, SimoneMatsugaki, AiraBenjamin, DonMatsumoto, KunioBentel, Jacqueline M.Mattoscio, DomenicoBerezovski, MaximMaugeri, MarcoBertacchini, JessikaMavila, NirmalaBerthelet, JeanMawhinney, ThomasBhanot, HaymantiMaye, Peter F.Bhosale, PriyaMcArdle, StephanieBi, YingtaoMcCann, AmandaBidère, NicolasMcCarthy, James B.Biedermann, ThomasMcGowan, EileenBigey, PascalMcGrath, EoghanBijlsma, Maarten F.McLeod, GeriBilen, Mehmet AsimMcVicker, BenitaBilger, AndreaMeckes, DavidBishehsari, FarazMedová, MichaelaBiswas-Fiss, Esther E.Meggetto, FabienneBittoni, MarisaMehrabi, ArianebBlackmore, PeterMehrpour, MaryamBlandino, G.Meldolesi, JacopoBlank, Christian U.Mendes, Ruheena L.Blasiak, JanuszMenendez, DanielBlonska, MarzennaMenendez, Sofia T.Boddicker, Rebecca L.Mercadante, SebastianoBoehme, KarlMeriggi, PaoloBoesch, MaximilianMertz, JanetBogani, GiorgioMeryet-Figuière, MatthieuBogdanov, MikhailMessaritakis, IppokratisBogni, AlessiaMesserli, Shanta M.Boichuk, SergeiMessner, DonaldBonnelye, EdithMezzanzanica, DeliaBontempo, PaolaMiceli, LucaBooth, BrianMichallet, Marie-CécileBora-Singhal, NamrataMichalopoulos, GeorgeBorchmann, SvenMicheau, OlivierBorrebaeck, Carl A. K.Michelle, PalmieriBorrego, FranciscoMichiels, CarineBossenmeier, BirgitMiddeldorp, Jaap M.Bottazzi, BarbaraMierke, Claudia TanjaBotti, GerardoMikropoulos, ChristosBouček, JanMilavetz, BarryBoulaiz, HouriaMilione, MassimoBourdon, Jean-ChristopheMiller, Anthony B.Bourgeois-Daigneault, Marie-ClaudeMiller, Jeffrey S.Bouvier, Anne-MarieMiller, KyleBowers, Laura W.Mills, KenBozec, AlineMinucci, AngeloBrady, GarethMisawa, KiyoshiBrahme, AndersMitra, AnirbanBrancaccio, MaraMitra, Siddhartha ShankarBrandsma, DietaMiwa, ShinjiBrcic, LukaMiyata, YasuyoshiBréchot, PatriziaMiyazaki, MasaruBreckpot, KarineMiyazawa, MasaakiBrekken, Rolf A.Mlejnek, PetrBrennan, PaulMo, Yin-YuanBrewer Gutierrez, OlayaMoccia, FrancescoBrim, HassanMock, BeverlyBrinckerhoff, Constance E.Modos, DezsöBrinkmann, MelanieModrowski, DominiqueBroccolo, FrancescoMoen, ErikaBrown, GeoffreyMoens, UgoBrown, JamesMoffitt, RichardBrugières, LaurenceMohamad, OsamaBrunnström, HansMohammad, Ramzi M.Brust, PeterMohammed, SulmaBryant, CurtisMöhlendick, BirteBuccisano, FrancescoMoise, AlexanderBuchsbaum, JeffreyMolenaar, Remco J.Buchwald, ChristianMoller, PalBuckhaults, Phillip J.Mologni, LucaBuddaseth, SalmaMoncayo, RoyBudhu, SadnaMonga, VarunBuijs, JeroenMongiat, MaurizioBurdette, Joanna E.Montazeri Aliabadi, HamidrezaBurger, MichaelMontgomery, McKaleBurger, RenateMontgomery, Nathan D.Burke, DermotMoore, KathleenBuscail, LouisMorales, JulioBush, David A.Moreno, Carlos S.Caba, OctavioMorgenstern, DanielCadamuro, MassimilianoMoriggl, RichardCaignard, AnneMorishita, KazuhiroCaisová, VeronikaMoriyama, MitsuhikoCaja, LaiaMoro, LoredanaCalapre, LeslieMoroishi, ToshiroCalorini, LidoMorris, DavidCalvisi, DiegoMorris, DonCampagna, SaraMorris, James L.Caocci, GiovanniMucignat-Caretta, CarlaCappelletti, VeraMudryj, MariaCarbone, CarmineMueller, SoerenCaretta, AntonioMueller, VolkmarCaria, PaolaMühlebach, Michael D.Carlomagno, ChiaraMukaida, NaofumiCarnero, AmancioMukhtar, HasanCarpenter, RichardMulder, FritsCarreras, JoaquimMuller, MartinCarter, WayneMuller, Patricia A. J.Castellón, Enrique A.Müller-Newen, GerhardCastriconi, RobertaMuñoz Caffarel, MaríaCastrillon, Diego H.Mupo, AnnalisaCastro-Vega, Luis JaimeMurata, TakayukiCatacuzzeno, LuigiMurphy, Daniel J.Cattaneo, FabioMurray Stewart, TracyCeranowicz, PiotrMurray, PaulCerchia, LauraMuscarella, Lucia AnnaCeresa, BrianMusso, OrlandoChaffer, Christine L.Mustacchi, GiorgioChakraborty, SayanMutis, TunaChan, Annie W.Nadiminty, NagalakshmiChang, Hsueh-WeiNagayama, YujiChang, HyunNakamura, KyoheiChang, Joe Y.Nakanishi, TakeoChang, Nan-ShanNakanishi, YokoChang, Wei-ChiaoNakashima, HiroshiChang, Wen-WeiNakayama, KohChantrill, Lorraine A.Nandi, SaikatChapman, Jocelyn S.Narayanan, RameshChapoval, Svetlana P.Nariai, TadashiChatterjee, IshitaNass, NorbertChatterjee, NimratNasser, MohdChaudhuri, NaziaNavran, ArashChauvie, StephaneNemoto, KiyomitsuChekenya, MarthaNephew, KennethChen, Mei-RuNeri, AntoninoChen, SuzieNeuhaus, JochenChen, WenchunNgan, Elly Sau-WaiChen, Wen-TienNguyen, Minhhuyen T.Chen, XiangNi, HeyuChen, ZhihangNibu, Ken-ichiCheng, ChunmingNice, EdouardCheng, HailiNielsen, Finn CiliusCheng, KeNierkens, StefanCheon, Dong-JooNieveen Van Dijkum, E.J.M.Chesi, MartaNilubol, NarisChevret, EdithNing, ShunbinChia, Jing-ShanNishina, HiroshiChiam, KarenNitulescu, George MihaiChiappinelli, KatherineNoble, SimonChien, JeremyNonami, AtsushiChinen, Ludmilla Thomé DomingosNorihiko, NaritaChiu, Ka Fung PeterNormanno, NicolaChmura, Steven J.Notarbartolo, MonicaCho, HyosunNoubissi, Felicite KamdemChobot, VladimirNowak-Sliwinska, PatrycjaChoi, HaesunNowotarski, ShannonChoi, Kyung-ChulNozaki, MasamiChoi, MinsigNuovo, Gerard J.Choudhary, VivekNylander, KarinChow, DanielO’Brien, Katie M.Choy, EdwinOdenthal, MargareteChu, Ching-LiangOgi, KazuhiroChu, DafengO’Hagan, Heather M.Chu, XiangpingOhno, HitoshiChun, Kyung-SooOhno, TatsuyaChung, JunhoOhtani, KiyoshiChung, VincentOkada, MasahiroCiaglia, ElenaOkajima, TetsuyaCiarrocchi, AlessiaO’Neill, EricCiccarelli, RenataOno, SatoshiCiccarese, ChiaraOrimo, AkiraCicone, FrancescoOrth, James D.Cifone, Maria GraziaOrtolano, SaidaCimmino, AmeliaOrzechowski, ArkadiuszCiribilli, YariOsaki, MitsuhikoCisowsky, JaroslawOse, JenniferClaing, AudreyOsera, CeciliaClark, Geoffrey J.Oshima, YusukeColey, Helen M.Ostrin, Edwin J.Collavin, LicioOttewell, Penelope D.Coller, Hilary A.Ove, RogerCollery, PhilippeOzsvari, BelaConfavreux, CyrillePabst, ThomasConibear, JohnPaggetti, JérômeConradi, Lena ChristinPaietta, ElisabethConstantinescu, StefanPalani, ChitraConway, Edward M.Palese, PeterCook, LeahPallante, PierlorenzoCooper, Arthur J. L.Paller, ChanningCordenonsi, MichelangeloPalmer, Christopher P.Cordo Russo, Rosalia I.Palumbo, PaolaCorey, SethPaluszczak, JarosławCorr, Stuart J.Pan, MingguiCorrenti, MargheritaPan, ZuiCorsi, FabioPanasyuk, GannaCortes-Dericks, LourdesPanda, AmareshCortesi, LauraPandit, HarshulCorvigno, SaraPaolini, FrancescaCosimelli, MaurizioPapa, AlfredoCostantini, SusanPapacleovoulou, GeorgiaCosta-Silva, BrunoPapageorgis, PanagiotisCotelle, PhilippePapoudou-Bai, AlexandraCrane, Courtney A.Parashar, DeepakCrawford, LindseyPark, Jae MoCrescioli, SilviaPark, JohnCrespan, EmmanuelePark, Jong KookCress, Anne E.Park, SerkinCroce, Anna CletaPark, Sung-hyeCrook, Zachary R.Park, Woo-YoonCroset, MartineParra-Guillen, Zinnia PatriciaCuburu, NicolasPatching, Simon G.Cyr, Daniel G.Patel, Sapna P.Czerniecki, BrianPaulsson, Johan O.Czyż, JarosławPeng, TengD’Elios, Mario MilcoPennock, NathanDahl, GerhardPenzo, MariannaDai, NingPeppelenbosch, MaikelDakhlallah, DuaaPero, RaffaelaDal Col, JessicaPerrais, MichaëlDalamaga, MariaPeruzzi, FrancescaDalin, Martin G.Petroff, Brian K.Damm-Welk, ChristinePezzella, FrancescoDanila, Daniel C.Pezzuto, AldoDann, Eldad J.Piazza, CesareDardenne, EtiennePiccolo, StefanoD’Argenio, ValeriaPiché, AlainDark, Michael J.Pierantoni, Giovanna MariaDaverey, AmitaPilarsky, ChristianDavis, Richard E.Pinzani, PamelaDavuluri, RamanaPirtoli, LuigiDe Luca, AntonioPitts, Todd M.De Nigris, FilomenaPiva, RobertoDe Re, ValliPiva, TerrenceDe Robertis, MariangelaPlaydon, MaryDe Rosa, VivianaPoff, Angela M.De Vita, AlessandroPoggi, AlessandroDe Vita, ValerioPolakowski, Nicholas J.De Wit, SannePolicastro, LuciaDeBerardinis, Ralph J.Pollok, Karen E.Decaestecker, ChristinePoma, PaolaDeckers, R.H.R. (Roel)Ponz-Sarvise, MarianoDecock, JuliePooler, DarcyDeep, GaganPoplawski, PiotrDeGregori, JamesPorunelloor, MathewDel Ben, FabioPosch, FlorianDel Sal, GianninoPosselt, GernotDelikatny, E. JamesPostovit, Lynne-MarieDelisle, Jean-SébastienPotenza, NicolettaDellinger, Thanh HuePouliot, NormandDellis, OlivierPourquier, PhilippeDelort, LaetitiaPowathil, GibinDelpon, GrégoryPowrózek, TomaszDemidov, ValentinPrestwich, Robin J. D.Demir, Ihsan EkinProsperi, JeniDer Schaff, Arjen VanPuglisi, FabioDeville, CurtilandPunganuru, Surendra ReddyDevilliers, Ethel-michelePunyadeera, ChamindieD’Haene, NickyPurrello, MicheleDhamija, SonamPutzke, AaronDharmawardhane, SuranganiePuxeddu, EfisioDharnidharka, VikasPyne, SusanDi Buduo, ChristianQian, GuoqingDi Fazio, PietroQueirolo, PaolaDi Tucci, Chiara Quintarelli, ConcettaDi Zazzo, ErikaRades, DirkDíaz-Carballo, DavidRahib, LolaDidonna, AlessandroRai, VikrantDiederich, MarcRaimondi, CristinaDíez Valle, RicardoRaj, NitinDimitrakopoulou-Strauss, AntoniaRajurkar, MihirDimitriadis, Emilios K.Ramani, KomalDimitroff, Charles J.Ramaraj, PanduranganDing, KaiRamasamy, MohankandhasamyDiniz, CarmenRamchandani, DivyaDinulescu, Daniela M.Ramirez-Garcia, AndoniDiPersio, MikeRamondetta, Lois M.Divella, RosaRampazzo, ElenaDjavaheri-Mergny, MojganRampias, TheodorosDoddapaneni, RaviRangaswami, ArunDomenici, GiacomoRanieri, ElenaDomschke, ChristophRapic, SaraDong, JixinRashidi, ArminDong, Xian-PingRatovitski, EdwardDonnelly, OliverRattan, RamandeepDou, Q. PingRattray, Nicholas J. W.Dowty, JamesRavindranath, AditiDrabkin, HarryRaz, VeredDrakes, Maureen L.Rebelo, Sofia P.Drillien, RobertRecalcati, StefaniaDrott, JennyRECHER, ChristianDu, WaRedell, MicheleDudas, JozsefRee, Anne HansenDuennwald, Martin L.Reinartz, SilkeDuffy, MargaretRemaut, KatrienDutil, JulieRenner, UlrichDutta, ArijitRescigno, AntonioDyer, MartinReynolds, PaulDziegielewska, BarbaraRha, Sun YoungDzimitrowicz, AnnaRibera, José MariaDzobo, KevinRicci, GiuliaEcke, ThorstenRich, Jeremy N.Eckert, FranziskaRichardson, AlanEdeline, JulienRichter, AntjeEfird, JimmyRiesterer, OliverEisinger, Tzipora Sarah KarinRinnerthaler, GabrielEke, IrisRiza, ElenaEkVitorin, JoseRizos, HelenEl Bairi, KhalidRizzo, MilenaEl-Heliebi, AminRizzolio, FlavioEllies, Lesley G.Roato, IlariaElshimali, Yahya I.Roberts, Arthur G.Emadi, AshkanRobles, Ana InesEmeto, Theophilus I.Roby, KathyEmfietzoglou, DimitrisRocchi, StéphaneEndersby, RaeleneRodrigo, Juan P.Endo, MakotoRodríguez-Aguayo, CristianEngeland, Christine E.Rodriguez-Lafrasse, ClaireEngeland, KurtRohr-Udilova, NataliyaEoli, MaricaRoidl, AndreasEpplein, MeiraRomani, MassimoErenpreisa, JekaterinaRoncucci, LucaEscargueil, AlexandreRoos, Wynand P.Eubank, TimothyRosado, Juan A.Eufemi, MargheritaRosato, AntonioEvrard, Solène M.Rosell, RafaelExcoffon, KatherineRosenberg, ShaiEykyn, Thomas R.Rössler, KarlFabelo, HimarRoszik, JasonFabiani, RobertoRouleau, EtienneFacchini, GaetanoRousselle, PatriciaFaehling, MartinRovida, ElisabettaFahraeus, RobinRowan, AileenFakhouri, Walid D.Rowther, Farjana B.Falasca, MarcoRuiz De Garibay, GorkaFan, AliceRuiz-Gómez, GloriaFan, MeiyunRuiz-Narváez, Edward A.Fang, XianjunRuiz-Pérez, María VictoriaFanizzi, Francesco PaoloRusinek, DagmaraFaraone Mennella, Maria RosariaRussell, RyanFarquhar-Smith, W. PaulRusso, AnnapinaFava, CarmenRzymowska, JolantaFedele, MonicaSabri, SihamFeng, YibinSadik, RiadhFeo, FrancescoSafa, Ahmad R.Ferrara, Marianna LucianaSaha, AshirbaniFerro, MatteoSaid, NeveenFerroni, PatriziaSaini, ShikhaFilleur, StephanieSainz Jr., BrunoFinlay-Schultz, JessicaSaito, AkiraFinocchiaro, GaetanoSaito, RyokoFiorio Pla, AlessandraSakaguchi, MasakiyoFisher, Jonathan S.Saki, NajmaldinFlorea, Ana-MariaSalam, AkbarFogarty, GeraldSalaspuro, MikkoFonseca, RafaelSalgado, TeresaForde, PatrickSalmon, IsabelleFornari, FrancescaSammartino, PaoloForte, EleonoraSanders, NiekFranceschini, DavideSantarelli, AndreaFrancis, HeatherSantolla, Maria FrancescaFranco, RenatoSantoni, GiorgioFranco-Barraza, JanuszSantoni-Rugiu, EricFrandsen, Stine KrogSara, ZalbaFranken, Nicolaas A. P.Satake, HironagaFrère, CorinneSato, KoFribley, Andrew M.Sato, TakamiFriedrich, ThomasSato-Bigbee, CarmenFries, Jochen W. U.Saule, SimonFriis-Hansen, LennartSavaşan, SüreyyaFroehner, MichaelSayers, Thomas J.Fröhlich, Leopold F.Scarpi, EmanuelaFu, PanfengSchartinger, VolkerFujihara, HisakoSchatz, Jonathan H.Fujii, Shin-ichiroSchemmer, PeterFujita, MitsuguSchlaepfer, Isabel R.Fujita, TetsujiSchlegel, JürgenFujita, YuSchönthal, Axel H.Fukayama, MasashiSchuller, Hildegard M.Fukuda, TakeshiSchulte, ReinhardFukumitsu, NobuyoshiSchultz, Kris Ann P.Funel, NiccolaSchulz, FlorianFuric, LucSchwartz, KennethFurukawa, TatsuhikoSchwertfeger, KayleeFurusawa, YukihiroSchwiebs, AnjaFuruya, HidekiScudiero, OlgaFuso, AndreaSeano, GiorgioFutakuchi, MitsuruSedic, MirelaGahete, Manuel D.Seidlits, StephanieGalati, RossellaSeipel, KatjaGaletti, MariclaSekido, YoshitakaGameiro, SofiaSemenov, IuriiGanau, MarioSen, MalabikaGandhi, Maher K.Seno, MasaharuGao, DingchengSenter, LeighaGao, ShuaiSersa, GregorGao, YangSeshacharyulu, ParthasarathyGarcía Fernández, AntoniaSeshadri, MukundGarcía-Jiménez, CustodiaShackelford, JuliaGarcia-Lora, Angel MiguelShahda, SafiGarcia-Rico, EduardoShair, Kathy H.Y.Gardiner, Clair M.Shakeel, ModakGarofalo, MariangelaShanmugam, Muthu K.Garvalov, Boyan K.Shao, HaipengGatzka, MartinaShao, Yu-YunGazouli, MariaSharan, ShyamGe, XiaodongSharma, Amar DeepGębicki, JacekSharma, DipaliGentile, VittorioSharma, GeetanjaliGentilini, DavideSheldrake, HelenGentner, BernhardShen, Jia ZackGeorgakilas, AlexandrosShen, Tang-LongGeorge, AngelaSherer, NathanGerlitz, GabiSherman, JonathanGertych, ArkadiuszShewach, DonnaGewirtz, David A.Shi, LewisGhislat, GhitaShi, WenyinGiacomelli, ChiaraShi, YangGiannattasio, SergioShi, ZhiGiantsoudi, DrosoulaShibata, YasushiGilbert, RichardShidal, ChrisGilles, ChristineShim, SupGillespie, TheresaShimodaira, ShigetakaGiordano, AntonioShin, Vivian YvonneGiovannetti, ElisaShinohara, ShogoGirard, Pierre MarieShiozawa, YusukeGismondi, AngelaShirakami, YoheiGisselbrecht, ChristianShirley, ShawnaGiusti, IlariaShnyder, SteveGiusti, RaffaeleShoshan, MariaGjertsen, BjörnSiddappa, ByrareddyGlass, Kimberly R.Siddiqui, ImranGlimelius, IngridSiena, SalvatoreGloor, BeatSignori, EmanuelaGodlewska, MarlenaSikkema-Raddatz, BirgitGoedert, JamesSikorski, AleksanderGoel, GauravŠikurová, LibušaGoel, PeeyushSill, HeinzGogoi, RadhikaSilva, Adriana RibeiroGolan, TaliaSilvestre, SamuelGoldberg, Gary S.Sim, TaeboGollin, Susanne M.Simmen, Frank A.Golubovskaya, VitaSinger, Eric A.González Granado, José MaríaSingh, AnilGonzález, Florenci V.Singh, BhagirathGonzález-González, RogelioSinnamon, Andrew J.Goodfellow, Paul J.Sitcheran, RaquelGorgoulis, VassilisSkubitz, Amy P.N.Gorini, FrancescaSlack, JamesGorman, AdrienneSlominski, AndrzejGouvas, NikolaosSmahel, MichalGovin, JérômeSmerage, Jeffrey B.Grand, Roger J.Sminia, PeterGrande, FedoraSmit, LindaGranucci, FrancescaSmith, Jill P.Grässer, FriedrichSmith, Sinead M.Gray, Elin S.Smolle, Maria A.Gray, Steven G.Solana, RafaelGreither, ThomasSoldevilla, MarioGriffin, RobertSoleymani Abyaneh, HodaGrignani, GiovanniSoloff, Adam C.Grimes, Mark L.Solov’yov, AndreyGrimm, ChristianSon, Deok-sooGrippo, Paul J.Song, ChunhuaGrivas, PetrosSong, HaiweiGrizzi, FabioSorrenti, ValeriaGrösch, SabineSottnik, JosephGross, ThomasSouyri, MicheleGryshkov, OleksandrSpina, ValeriaGudey, Shyam KumarSpizzo, RiccardoGuerra, CarmenSpratt, DanielGuillaumond, FabienneSprenger, CynthiaGuillermet Guibert, JulieSprio, Andrea ElioGuix, Ester BallanaSrivastava, AkhilGullberg, DonaldSrivastava, Pramod K.Gulley, MargaretSrivatsan, Eri S.Guo, SiqiStack, M. SharonGuo, WeiminStanley, Jone A.Guo, YanStarr, Timothy K.Guo, Zong ShengSterling, JulieGupta, SubashSterpin, EdmondGuttery, David S.Stewart, Lamonica V.Ha, PatrickStiller, Carl-OlavHackshaw, AllanStoehr, RobertHaendler, BernardStorti, PaolaHafizi, SassanStoven, VéroniqueHagemann, CarstenStrano, SabrinaHalama, AnnaStrigari, LidiaHall, MarciaStrojan, PrimožHallberg, BengtStudebaker, Adam W.Haluza, DanielaSu, Wu-ChouHamanishi, JunzoSubbiah, VivekHamblin, MichaelSuda, KenichiHamilton, GerhardSudhir, Putty ReddyHammer, ElkeSudol, MariusHan, DongSugikawa, KoutaHannah, OhSugimoto, M.Hansen, Adam E.Sugimoto, NaotoshiHanson, Lars G.Suh, K. StephenHaque, InamulSuh, Pann-GhillHarada, HisashiSukocheva, OlgaHarada, MamoruSun, LichunHarn, Horng-JyhSun, XuezhengHarris, Reuben S.Sun, Yu-tingHarrison, Jonathan S.Sunaga, NoriakiHartman, MariuszSunami, YoshiakiHaslene-Hox, HanneSung, PatrickHassan, MohamedSurdutovich, EugeneHasselbalch, HansSusi, PetriHatachi, YukimasaSuzui, MasumiHaupt, YgalSuzuki, HiroyukiHawkins, Shannon M.Suzuki, KeijiHayashi, RyujiSwierczynski, JulianHazawa, MasaharuSzabò, IldikòHeckman, CarolineSzegezdi, EvaHeemers, HanneloreSzeliga, MonikaHehlgans, StephanieSzewczuk, Myron R.Hendriks, LizzaTabernero, María DoloresHenson, AdriannaTafur, Alfonso JavierHerold, NikolasTagaya, YutakaHiggins, Paul J.Tajima, TsuyoshiHildreth, BlakeTakahashi, HirokiHildt, EberhardTakamatsu, ShigeyukiHirsch, EmilioTakashi, ImaiHisada, YoheiTakayama, KenichiHishikawa, YoshioTakebe, NaokoHoang-Vu, CuongTakenaka, YukinoriHochwald, Steven N.Takyar, SeyedtaghiHodivala-Dilke, KairbaanTamae, DanielHoffman, RobertTan, Shyh-HanHoffmeister, MichaelTan, WinstonHohaus, StefanTanaka, NobuyukiHøivik, Erling AndreTandra, Pavan KumarHolland, Angelia MaleahTang, JohnnyHollevoet, KevinTao, JunyanHompland, TordTarallo, ValeriaHong, In-SunTartour, EricHooda, JagmohanTashiro, EtsuHori, ToshiyukiTassone, PierfrancescoHoskins, PaulTateishi, KensukeHourigan, ChristopherTerracciano, Carlo ManciniHsieh, Chia-HungTesta, UgoHsieh, James J.Tezil, TugsanHsu, Kuo-ShengTham, IvanHsu, Sung-PoTheilmann, LorenzHu, Dong GuiThomas, ChristoforosHu, GuangzhenThomas, Seddon Y.Hu, WenweiThomas, SufiHuang, LeiThomas, T. J.Huber, PeterThompson, BarryHuczyński, AdamThorat, NanasahebHuebbers, Christian U.Thuringer, DominiqueHug, EugenTinsley, Sara M.Hui, Kwai FungTiriveedhi, VenkataswarupHumar, MatjazTogo, ShinsakuHung, Jan-jongToh, Tan BoonHuppi, KonradToivanen, RoxanneHur, HoonToivola, DianaHusnjak, KoraljkaTokino, TakashiHutchison, Robert E.Toledo, FranckHuwiler, AndreaTomaić, VjekoslavHwa Soung, YoungTomasetti, MarcoHwang, Wei-LunTommasino, FrancescoHyde, R. KatherineTommasino, MassimoHyun, Kim TaeTomuleasa, CiprianIbrahim, Sherif AbdelazizTornesello, MariaIdbaih, AhmedTorres, GabrielaIgaz, PeterTosi, SabrinaIkeda, FusaoToss, AngelaIlmer, MatthiasToth, ZsoltIm, Chang-NimTownsend, AmandaImai, YoichiTraer, ElieImig, JochenTrajkovic, VladimirImperlini, EstherTramontano, DonatellaInamura, KentaroTrenti, TommasoInfante, PaolaTrepanier, AngelaInga, AlbertoTrinchera, MarcoInoue, KazushiTripathi, ManishIsidoro, CiroTrivedi, PankajIturri, JagobaTrobaugh-Lotrario, AngelaIyer, RenukaTroncone, GiancarloIzadifar, ZohrehTrosko, James EdwardIzasa, HisashiTsai, C. H.Izumiya, YoshihiroTsai, Ming-ShaoJabbari, BahmanTsang, Kwong YokJanakiraman, HarinarayananTsapogas, PanagiotisJanecki, MarcinTsoulfas, GeorgeJang, Sung-WukTsuchiya, HiroyukiJaniszewska, MichalinaTu, ThomasJansen, MarnixTullo, ApolloniaJanuchowski, RadoslawTuretta, MatteoJaved, MahmoodTurley, Eva A.Jazaeri, AmirTurner, SuzanneJazirehi, Ali R.Tuyaerts, SandraJean, ChristineTwu, Yuh-ChingJean, DidierTyne, Angela L.Jeffery, PennyTzanakakis, George N.Jelena, KrsticUchida, ShizukaJemielity, JacekUhl, EberhardJenkins, IanUlasov, IlyaJenkins, Samir V.Ulivi, PaolaJeong, Keun-YeongUmemura, AtsushiJeremić, BranislavUpham, Brad L.Jeremy, Teoh Yuen ChunUrbanelli, LorenaJeronimo, CarmenUsui, TatsuyaJeschke, UdoValentovic, MonicaJetten, AntonValle, LauraJia, DongyaVan De Vijver, KoenJia, XunVan Den Bergh, HubertJiang, HongVan Den Beuken-van Everdingen, M. H. J.Jiang, JianxiongVan den Brekel, Michiel Wilhelmus MariaJiang, Lin-HuaVan Der Wekken, AnthonieJiang, SizunVan Doorslaer, KoenraadJiang, YanyanVan Gent, Dik C.Jin, XinVan Noorden, CornelisJo, Young SukVan Raay, TerryJonckheere, NicolasVan Waardenburg, Robert C. A. M.Jones, Richard J.VanBrocklin, MatthewJoseph, RichardVanderhyden, BarbaraJoubert, NicolasVarki, Nissi M.Ju, XinshengVaron, ChristineJuhlin, Carl ChristoferVasko, VasylJung, Alain C.Vassetzky, YegorJung, Chan KwonVavvas, Demetrios G.Jung, KlausVeeramani, SureshJunker, SteffenVega, FranciscoKabała-Dzik, AgataVelasco, EladioKagey, Jacob D.Veltkamp, ChristianKageyama, YukioVenkitachalam, SrividyaKahn, MichaelVenturella, RobertaKaigorodova, Evgeniya V.Venza, MarioKairemo, KaleviVergara, DanieleKajiya, HiroshiVermeer, Paola D.Kakar, ShamVezzoni, PaoloKallergi, GalateaViapiano, Mariano SebastianKamada, TadashiVictoria, El-KhouryKamaleddin, Mohammad AminViel, SébastienKamato, DanielleVillalobos, CarlosKandimalla, RajuVillasante, AranzazuKane, EleanorVinciguerra, ManlioKang, Chang MooViola, GiampietroKang, WeiVisser, LydiaKango-Singh, MadhuriVitale, MassimoKao, Shao-HsuanVoelkers, MirkoKao, Shu-HueiVogt, AndreasKappler, MatthiasVranic, SemirKaramitopoulou, EvaWada, JunKarayiannakis, AnastasiosWain-hobson, SimonKashiwagi, ShinichiroWalters, ZoeKashuba, ElenaWang, ChengKasinski, AndreaWang, EuniceKasman, LauraWang, GuozhengKaspi, EliseWang, KaiKastrati, IridaWang, ManKatakura, AkiraWang, RanranKatapodi, Maria C.Wang, RuiKato, Takamitsu A.Wang, WenqiKato, YasumasaWang, YulingKatoh, HironoriWang, Yun-MingKawasumi, MasaokiWarkiani, Majid EbrahimiKawauchi, KeikoWatanabe, MasatoshiKe, YouqiangWatson, PhilipKemp, Michael G.Weaver, AlissaKensler, Thomas W.Weber, Damien C.Kerr, BethanyWebster, Marie R.Khalil, Ahmad MohammadWei, ChangyongKhanim, FarhatWei, YonglongKhanna, RajivWeigand, Julia E.Khoo, Bee LuanWellner, UlrichKiejda, Kelly A. AveryWelner, RobertKikuchi, EijiWelters, Hannah J.Kim, Byung SooWestover, KennethKim, E. EdmundWhelan, RebeccaKim, Hyo SongWhitehurst, ChristopherKim, Jung-AeWicklein, DanielKim, Raymond H.Wieczorek, EdytaKim, RichardWiemer, Erik A.C.Kim, Sang-WeWikman-Kocher, HarrietKim, Sung-HoonWikström, PernillaKim, Tae-HeeWilley, Christopher D.Kim, TaewanWilliams, BrianKim, Won SeogWilliams, Stephen J.Kim, Yong-miWilmink, Johanna W.Kimpel, JanineWilson, EricaKing, TamaraWilting, JorgKlampfer, LidijaWinkler, EvaKlapdor, RüdigerWise, Petra M.Klebe, SonjaWondrak, Georg T.Kleeff, JörgWong, DavidKlement, RainerWong, Kwong-KwokKlinke, DavidWong, Lee-Jun C.Knecht, HansWong, Stephen T. C.Koistinen, HannuWong, SunnyKoizume, ShiroWorth, Randall G.Kong, MoonkyooWu, AlexanderKong, YimengWu, Yu-shanKoo, Ja SeungWu, Zhao-HuiKoom, Woong SubXia, BingKopechek, JonathanXia, ShuliKorkaya, HasanXiang, DongxiKornprat, PeterXie, JingwuKosinsky, Robyn LauraXie, PingKovács, AttilaXu, JieKovács, GyörgyXu, YanKoval, AlexeyYamada, TadaakiKoyama, YuYamamoto, MasatoKozlowski, PiotrYamamoto, YusukeKreeger, PamelaYamamura, SoichiroKretsovali, AndronikiYamaoka, ToshimitsuKrishna, Somashekar G.Yamaoka, YoshioKrol, SilkeYamazaki, EtsukoKrug, SebastianYan, BowenKrzykawska-Serda, MartynaYan, Catherine T.Kubo, ToshioYan, ChunhongKulangara, KarinaYan, DongqingKulasinghe, AruthaYang, DaQingKulawiak, BoguszYang, HushanKundrat, PavelYang, JunKunjachan, SijumonYang, KunKuno, HirofumiYang, LiliKuno, ToshiyaYang, LingLiKunz, MeikYang, WancaiKunzelmann, KarlYano, HajimeKurelac, IvanaYao, JiayiKurenova, Elena V.Yao, SongKuryk, LukaszYap, JasonKveberg, LiseYe, HaobinKwok, Wai-MengYe, XiaohuaKwong, Lawrence N.Ye, YanqiKypta, Robert M.Yeh, Elizabeth S.La Rocca, Renato V.Yeo, SynLa Vecchia, CarloYeung, Tsz-LunLaboisse, Christian L.Yi, TaoLaghi, AndreaYin, CameronLahooti, HooshangYing, HaoqiangLai, KeaneYochum, Gregory S.Lakshmanan, ImayavarambanYoneda, ToshiyukiLamar, John M.Yoon, Hyeun JoongLambertini, MatteoYoon, IlLamprou, DimitriosYoshida, KatsunoriLandry, JosephYoshiyama, HironoriLang, JulieYoshizaki, TomokazuLang, LiweiYoshizawa, AtsushiLangdon, Simon P.You, LiangLanger, FlorianYou, ZongbingLanger, RupertYoung, ArabellaLangner, CordYoung, ColinLara-Otero, KarlenaYoung, Howard A.Larijani, ManiYoung, JoanneLarsen, Anders ChristianYoung, M. Rita I.Lasfar, AhmedYoung, TravisLathia, Justin D.Yount, Jacob S.Laukkanen, MikkoYu, Cheng-PingLaurent, CamilleYu, DiLauriola, MattiaYu, Yan PingLaustsen, ChristofferYu, YanlinLavasanifar, AfsanehYuba, EijiLayne, Tracy M.Yue, JunmingLazarević, VladimirYura, YoshiakiLeanza, LuigiZagotto, GiuseppeLebleu, ValerieZahnow, Cynthia AnnLeclercq, GuyZamarchi, RitaLee, Che-HsinZani, Bianca MariaLee, Chong-KilZannini, MariastellaLee, Dae HoZebisch, ArminLee, HoZhang, HuiLee, Hsin-ChenZhang, Jian-minLee, JandeeZhang, JunLee, Jeong-HwaZhang, JunranLee, Joo HyoungZhang, WeihuaLee, Jun HeeZhang, WencaiLee, Jun SikZhang, YeLee, Kin Wah TerenceZhang, YunkaiLee, Kwang HyuckZhao, BinLee, NohyunZhao, HongyunLee, Sang-HanZhao, JiheLee, So YeongZheng, YiLee, SookyungZhivotovsky, BorisLee, WeiZhou, DawangLefebvre, TonyZhou, Heshan SamLeiphrakpam, Premila D.Zhou, JiangbingLemay, GuyZhou, WenchaoLemke, JohannesZhou, YunliLenardon, MeganZhu, HuaLeng, RogerZhu, MingyaoLengerke, ClaudiaZhu, XiangdongLeón, JosefaZhu, YuwenLeone, AntonellaZimmer, JacquesLeporatti, StefanoZmijewski, MichalLewinska, AnnaZoratti, MarioLewis, StephenZuco, ValentinaLeyland-Jones, BrianZüllig, RichardLi, ChiZustovich, Fable

